# Towards pathophysiology-based interventions for children with ADHD and increased screen time utilisation

**DOI:** 10.1016/j.ebiom.2022.104075

**Published:** 2022-05-21

**Authors:** Samuele Cortese

**Affiliations:** a Centre for Innovation in Mental Health (CIMH), School of Psychology, Faculty of Environmental and Life Sciences, University of Southampton, Highfield Campus, Building 44, Southampton SO17 1BJ, UK; b Solent NHS Trust, Southampton, UK; c Hassenfeld Children's Hospital at NYU Langone, New York University Child Study Center, New York City, New York, USA; d Division of Psychiatry and Applied Psychology, School of Medicine, University of Nottingham, Nottingham, UK

Attention-Deficit/Hyperactivity Disorder (ADHD) is the most common neurodevelopmental condition, affecting around 5-7% of school-aged children^1^. Compared to individuals without ADHD, those with ADHD are at higher risk of mental health disorders (such as anxiety or depression), physical conditions (including obesity and asthma), and maladaptive/risky behaviours (e.g., unintentional physical injuries or suicidal behaviours)^1^. An emerging body of evidence suggests that ADHD is also significantly associated with increased screen time utilisation (STU) (e.g.,^2^), which can add to the burden of ADHD. However, current clinical guidelines^3^ do not include any specific recommendation for the screening and management of increased STU in individuals with ADHD.

The study by Yang et al.^4^ furthers our understanding on the relationship between ADHD symptoms and STU, paving the way for possible pathophysiology-based interventions. The authors focused on polygenic risk scores (PRS), i.e., the sum of the effects of individual genes that provides an overall estimate of genetic lability for a given disorder. Elegantly leveraging data from more than 11,000 children from the Adolescent Brain Cognitive Development cohort, Yang et al. found that higher PRS for ADHD were significantly associated with longer STU, and that fractional anisotropy (FA, a magnetic resonance imaging measure that estimates white matter integrity) in tracts mainly involved in visual functions was negatively correlated with both the ADHD PRS and STU. Furthermore, FA values at baseline were significantly correlated with ADHD scores at 2-year follow-up and there was a bi-directional association between ADHD scores and STU at baseline and 1-year follow-up. Finally, FA scores and ADHD symptoms severity mediated the relationship between ADHD PRS and STU.

Do the findings by Yang and colleagues imply that children should be systematically screened via PRS to detect those who will likely develop ADHD and increased STU? The simple answer is no. Technically, PRS are defined by the weighted sum of the risk alleles which are associated with a disorder based on data from available Genome Wide Associations Study (GWAS). Currently, due to small effect sizes, ADHD PRS cannot accurately predict individual outcome. In other terms, most of those with high scores on the currently available ADHD PRS will not develop ADHD because “the signal is too weak”^5^. This is in line with what has been found for other conditions. For instance, less than 16% of individuals in the top decile of the PRS for obesity developed severe obesity at 25 year-follow-up, compared to around 1% in the bottom decile^6^.

So, where does the value of the study by Yang et al. lie? I believe it is in the understanding of the links between ADHD symptoms and STU at different levels, and in the implications for treatment development. By using a multimodal approach with a cross-sectional as well as longitudinal approach, the authors were able to build and test a comprehensive, coherent model linking ADHD and increased STU, spanning from genes to behaviour via brain measures (estimated white matter integrity). In turn, this points to possible intervention strategies. Indeed, there may be several reasons underlying the link between ADHD and STU. For instance, increased STU might stem from the behavioural impulsivity of individuals with ADHD, or increased STU could contribute ADHD symptoms, in particular inattention. However, the study by Yang et al. suggests that both ADHD and long STU share common genetic underpinning and are characterised by possible dysfunctions in white matter tracts related to visual functions. This may result in impaired executive control of visual functions, with enhanced sensitivity to and distractibility by visual stimuli leading to increased STU.

If replicated in other studies, the findings by Yang et al., would provide a compelling rationale to assess interventions aimed at restoring or improving these impaired visual functions. To date, the paradigm “gene dysfunction leading to brain imaging alterations, in turn underpinning behavioural manifestations” has not led to relevant innovations in the care of children with ADHD. For instance, meta-analytic evidence^7^ based on trials of cognitive training, aimed at changing the functioning of brain areas thought to mediate the relationship between genes and behaviour, has not supported the efficacy of this intervention in terms of improvement of ADHD core symptoms. This is unfortunate because, whilst stimulants have been shown to be highly efficacious - at least in the short term- for the core symptoms of ADHD^8^, their effects in terms of improving associated neuropsychological dysfunctions have been found to be less strong^9^. One possible reason is that individuals with ADHD included in such trials were highly heterogeneous in terms of underlying pathophysiological correlates. By adopting a precision psychiatry approach^10^ and selecting more homogeneous subgroups, such as children with high levels of ADHD symptoms and STU, the field might be more successful in designing and implementing novel pathophysiology-based interventions.

## Declaration of interests

SC declares honoraria and reimbursement for travel and accommodation expenses for lectures from the following non-profit associations: Association for Child and Adolescent Central Health (ACAMH), Canadian ADHD Alliance Resource (CADDRA), British Association of Pharmacology (BAP), and from Healthcare Convention for educational activity on ADHD.
